# A systematic review on maternal-to-infant transfer of drugs through breast milk during the treatment of malaria, tuberculosis, and neglected tropical diseases

**DOI:** 10.1371/journal.pntd.0011449

**Published:** 2023-07-13

**Authors:** Francis Williams Ojara, Aida N. Kawuma, Catriona Waitt

**Affiliations:** 1 Infectious Diseases Institute, Makerere University College of Health Sciences, Kampala, Uganda; 2 Department of Pharmacology and Therapeutics, Gulu University, Gulu, Uganda; 3 Department of Pharmacology and Therapeutics, University of Liverpool, Liverpool, United Kingdom; Broad Institute Harvard: Broad Institute, UNITED STATES

## Abstract

**Background:**

Exclusive breastfeeding of infants under 6 months of age is recommended by the World Health Organization. In 2021, over 300 million combined incident cases of malaria, tuberculosis, and neglected tropical diseases (NTDs) were reported, predominantly in low-income countries. For many of the drugs used as first-line treatments for these conditions, there is limited knowledge on infant exposure through breastfeeding with poorly understood consequences. This review summarized available knowledge on mother-to-infant transfer of these drugs to inform future lactation pharmacokinetic studies.

**Methodology:**

A list of first-line drugs was generated from the latest WHO treatment guidelines. Using standard online databases, 2 independent reviewers searched for eligible articles reporting lactation pharmacokinetics studies and extracted information on study design, participant characteristics, and the mathematical approach used for parameter estimation. A third reviewer settled any disagreements between the 2 reviewers. All studies were scored against the standardized “ClinPK” checklist for conformity to best practices for reporting clinical pharmacokinetic studies. Simple proportions were used to summarize different study characteristics.

**Findings:**

The most remarkable finding was the scarcity of lactation pharmacokinetic data. Only 15 of the 69 drugs we listed had lactation pharmacokinetics fully characterized. Most studies enrolled few mothers, and only one evaluated infant drug concentrations. Up to 66% of the studies used non-compartmental analysis to estimate pharmacokinetic parameters rather than model-based compartmental analysis. Unlike non-compartmental approaches, model-based compartmental analysis provides for dynamic characterization of individual plasma and breast milk concentration-time profiles and adequately characterizes variability within and between individuals, using sparsely sampled data. The “ClinPK” checklist inadequately appraised the studies with variability in the number of relevant criteria across different studies.

**Conclusions/significance:**

A consensus is required on best practices for conducting and reporting lactation pharmacokinetic studies, especially in neglected diseases such as malaria, tuberculosis, and NTDs, to optimize treatment of mother–infant pairs.

## Author summary

Malaria, tuberculosis, and neglected tropical diseases (NTDs) are prevalent in regions where exclusive breastfeeding is the norm and jointly contributed to more than 300 million incident cases in 2021 alone. Transfer of drugs from the mother to the breastfed infant through breast milk is poorly characterized for most drugs, especially those used for neglected conditions. The clinical consequences of drug exposure such as occurrence of toxicity or predisposition to antimicrobial resistance in the breastfed infants are poorly understood. Infant drug exposure is often estimated from maternal breast milk exposure rather than measurement of infant plasma. In our study, we summarized all existing data on breastmilk transfer of first-line drugs for malaria, tuberculosis, and NTDs. Very importantly, we have highlighted the worrying lack of knowledge and emphasized pertinent study design and data analysis aspects required to improve the accurate conduct and interpretation of lactation pharmacokinetic studies.

## Introduction

Malaria, tuberculosis, and neglected tropical diseases (NTDs) are among the leading causes of morbidity and mortality globally. The World Health Organization (WHO) estimated global incidence of malaria in 2020 was 241 million, with 627,000 malaria-related deaths [1]. In the same year, 10 million new tuberculosis infections and 1.5 million tuberculosis-related deaths were estimated [1]. NTDs are a diverse group of 20 conditions mainly prevalent in tropical areas, leading to an estimated 200,000 deaths and the loss of 19 million disability-adjusted life years, respectively, annually [1]. These diseases disproportionately affect poor communities and are significant contributors to the disease burden in sub-Saharan Africa and Southeast Asia.

WHO recommends artemisinin-based combination therapies (ACTs) as first-line for the treatment of uncomplicated malaria (except in pregnant women in their first trimester) [1]. Artemisinin derivatives have short plasma half-lives (approximately 2 hours) and are often coformulated with drugs such as lumefantrine or piperaquine, which have significantly longer half-lives to maintain an antimalarial effect long after the artemisinin derivative has been eliminated [2]. For the treatment of severe malaria, ACTs and quinine are recommended [1].

Tuberculosis is treated using antibiotic combinations extending 6 to 12 months. Rifampicin (R), Isoniazid (H), Pyrazinamide (Z), and Ethambutol (E) are the cornerstone for treatment of drug-sensitive tuberculosis. First-line treatment in new patients with pulmonary tuberculosis comprises 2 months with HRZE followed by 4 months with HR [3]. In rifampicin-susceptible and isoniazid-resistant tuberculosis, isoniazid is replaced by levofloxacin in a 6-month treatment. Up to 12 additional drugs, including moxifloxacin, bedaquiline, and linezolid are used in different combinations for treatment of multidrug-resistant tuberculosis [3].

NTDs are treated using a diverse range of drugs. Some common antimicrobial agents are used, for example, clarithromycin for the treatment of Buruli ulcers [1] and rifampicin for the treatment of leprosy [1]. For many other NTDs, older and highly toxic drugs are still widely used; for example, melarsoprol, discovered 73 years ago, is still the drug of choice for treating African trypanosomiasis (sleeping sickness) [4]. In some situations, such as for the parasitic infections leishmaniasis and onchocerciasis, treatment and eradication strategies involve the use of mass drug administration campaigns in which an entire population in a geographical location is treated regardless of whether they are infected or not.

**Fig 1:**
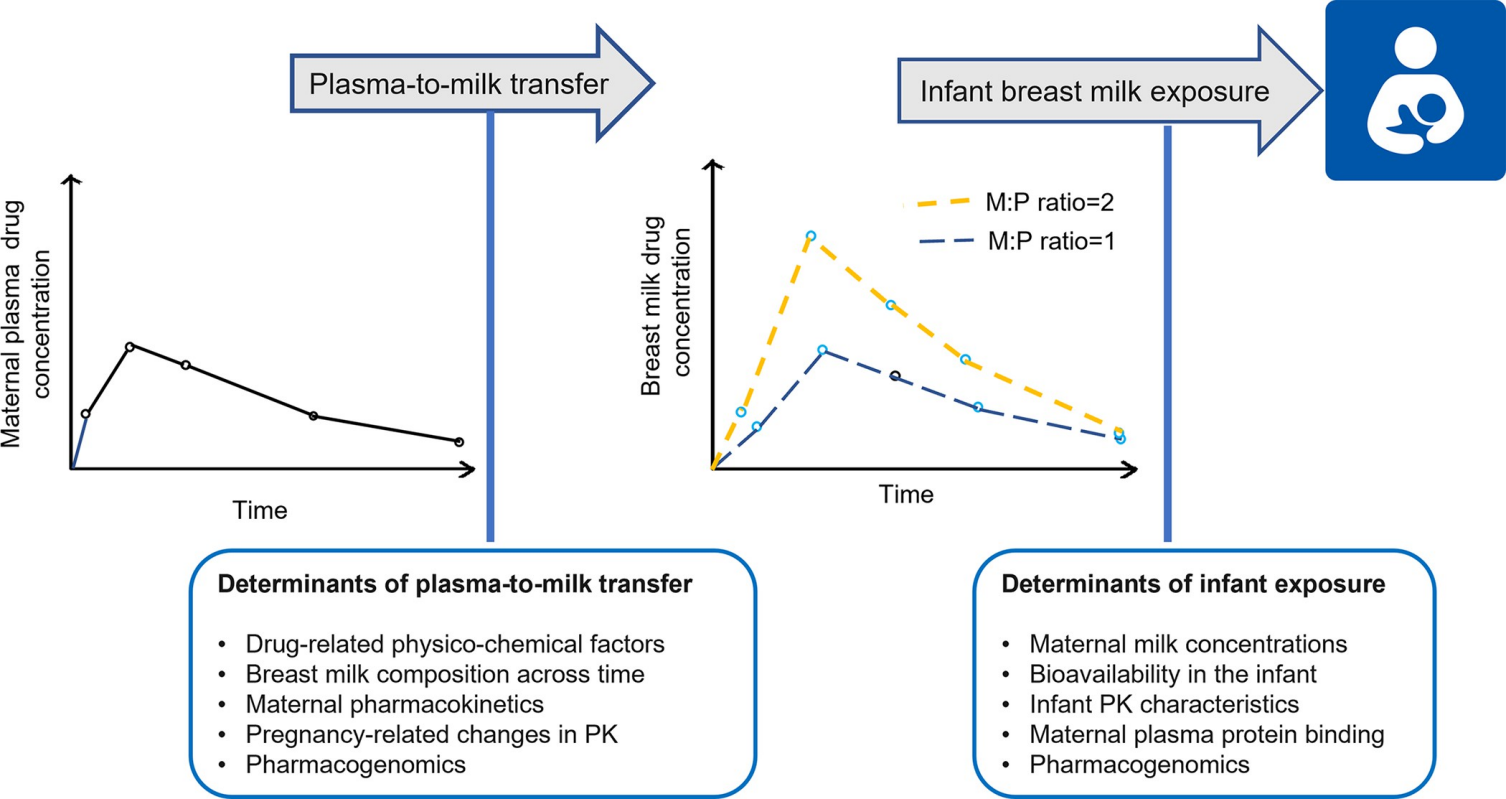
Schematic of maternal-to-infant transfer of drugs showing routinely estimated pharmacokinetic exposure factors and different factors that affect infant exposure (Clipart image obtained from https://openclipart.org/search/?query=breastfeeding+mother; date cited: May 30, 2023).

WHO recommends exclusive breastfeeding in the first 6 months of life, followed by a combination of breastfeeding and complementary foods [1]. Breast milk is a nutritionally rich matrix containing several micro- and macronutrients and is essential for child nutrition, development, and survival [1,5]. Many mothers take medication while breastfeeding, and where studies have been performed, most drugs found in the maternal blood have been found to be excreted in breast milk to some extent [6,7]. Infant exposure to drugs through breast milk depends on several factors including drug-related physicochemical properties, maternal physiology, and infant feeding patterns (**Fig 1**). A paucity of information on maternal-to-infant transfer of drugs may result in either (i) withholding prescription or choice not to take prescribed medication leading to interruption or undertreatment of maternal conditions; (ii) withholding breastfeeding, which disrupts its benefits for both mother and infant; and (iii) continuing breastfeeding and potentially exposing the infants to small doses of medication [6]. The clinical consequences of an infant’s exposure to drugs used to treat malaria, tuberculosis, and NTDs through breast milk are not clear. Possibilities include adverse reactions, protective exposure, and selection for resistance due to subtherapeutic exposure.

The ratio of drug concentration in maternal breast milk to that in maternal plasma (i.e., the milk-to-plasma [M:P] ratio) provides a crude estimate for infant exposure. The actual infant exposure depends on the M:P ratio, dose and dosing intervals, rate of plasma clearance, and the infant’s feeding patterns [8]. The relative infant dose (RID), calculated as a ratio of the daily infant dose to the daily maternal dose, provides a standardized relationship between maternal and infant exposure [9,10]. A theoretical infant milk intake of 150 mL/kg/day is widely used while calculating the RID; however, a dynamic, age-adjusted, infant milk intake model has previously been developed [11]. A RID >10% has been considered the threshold that should raise concern [12] and is widely cited, though this *widely accepted gold standard* does not account for the inherent toxic potential of the drug and, as a threshold, does not represent an exposure level at which all drugs can be considered toxic. RID calculations may also be based on breast milk volumes and concentrations obtained by completely emptying both breasts over a 24-hour period or by weighing the infant before and after every feed over 24 hours. However, the 2 approaches are not precise and are inconvenient for the mother and infant due to the multiple breast milk collections across 24 hours [13]. M:P ratio and RID are the primary parameters estimated in lactation pharmacokinetic studies. The informativeness of these studies depends on factors such as the number of mother–infant pairs enrolled, the number of samples collected per participant, and the statistical approach for data analysis.

The objectives of this systematic review were to (i) summarize all knowledge available on mother-to-infant transfer of drugs used to treat malaria, tuberculosis (drug-sensitive and rifampicin-resistant/multidrug-resistant, RR/MDR) and NTDs through breastfeeding and (ii) summarize the different mathematical approaches used to characterize maternal-to-infant transfer of drugs used to treat malaria, tuberculosis, and NTDs, to generate evidence on infant exposure and provide recommendations on analysis of lactation pharmacokinetic data for improved characterization of the exposure in a breastfed infant.

## Methods

### Drugs used to treat malaria, tuberculosis, and NTDs

Using the most recent WHO treatment guidelines for malaria, tuberculosis (including RR/MDR) and NTDs, we generated a list of drugs used to treat each of the respective diseases (Table 1). For the NTDs, up to 15 diseases were considered based on the WHO’s classification [1]. These include Buruli ulcer, Chagas disease, echinococcosis, foodborne trematodiases, human African trypanosomiasis (sleeping sickness), leishmaniasis, leprosy, lymphatic filariasis, mycetoma, chromoblastomycosis and other deep mycoses, onchocerciasis (river blindness), scabies, schistosomiasis, taeniasis/cysticercosis, and yaws and other endemic treponematoses. Other NTDs like rabies, envenoming due to snakebites, Guinea worm infections, dengue, and chikungunya were excluded because they lack specific drugs for their treatment. Once we compiled the list of drugs, we undertook a literature search for pharmacokinetic information on transfer into breast milk.

**Table 1:** World Health Organization (WHO)-recommended drugs for the treatment of malaria, tuberculosis (including RR/MDR), and neglected tropical diseases (NTDs).

Disease	Recommended treatments
Malaria [1]	**Uncomplicated malaria**: Artemether, artesunate, lumefantrine, amodiaquine, mefloquine, dihydroartemisinin, piperaquine, sulfadoxine-pyrimethamine, pyronaridine, atovaquone-proguanil, chloroquine, doxycycline **Severe (complicated) malaria:** Artesunate (IM or IV), quinine ***P*. *Vivax* or *P*. *Ovale* malaria**: Primaquine **Pregnant women in the first trimester:** Quinine, clindamycin
Tuberculosis [3]	**Drug-susceptible tuberculosis (patients new on treatment**): Rifampicin, isoniazid, pyrazinamide, ethambutol **Rifampicin-resistant/multidrug-resistant tuberculosis** Kanamycin, capreomycin, levofloxacin, streptomycin, moxifloxacin, bedaquiline, linezolid, clofazimine, delamanid, meropenem, imipenem-cilastatin, amikacin, p-aminosalicyclic acid, clavulanic acid, ethionamide, prothionamide
Buruli ulcers [1]	Rifampicin, clarithromycin, moxifloxacin
Chagas disease [16]	Benznidazole, nifurtimox
Echinococcosis [17]	Albendazole, mebendazole, praziquantel
Trematodiases [16]	Triclabendazole, praziquantel
Human African trypanosomiasis (Sleeping sickness) [4]	*T*. *b*. *gambiense* first stage: Pentamidine, fexinidazole *T*. *b*. *gambiense* second stage: Melarsoprol, erflonithine *T*. *b*. *rhodesiense* first stage: Suramin *T*. *b*. *rhodesiense* second stage: Melarsoprol
Leishmaniasis [18]	Sodium stibogluconate or meglumine antimonate, amphotericin B, miltefosine, paromomycin, pentamidine
Leprosy [1]	Rifampicin, clofazimine, dapsone
Lymphatic filariasis [19]	Ivermectin, albendazole, diethylcarbamazine citrate
Mycetoma [20]	Trimethoprim-sulfamethoxazole, streptomycin, isoniazid, rifampicin, minocycline, amikacin sulfate, amphotericin B, posaconazole, voriconazole, itraconazole
Chromoblastomycosis [21]	5-fluorocytosine, 5-fluorouracil, thiabendazole, amphotericin B, ketoconazole, fluconazole, posaconazole, terbinafine
Onchocerciasis (river blindness) [22]	Ivermectin
Scabies [23]	Ivermectin
Schistosomiasis (Bilhazia) [24]	Praziquantel
Taeniasis/cysticercosis [25]	Praziquantel, albendazole, niclosamide
Yaws [26]	Azithromycin, benzathine penicillin

### Literature search strategy

Using the online databases, PubMed, LactMed^®^, and SCOPUS, we performed a search using relevant key search terms (“Lactation” or “Breast milk” or “breast feeding” and “pharmacokinetics”) for each drug. The above online databases were searched for relevant studies by 2 independent reviewers (F. W. O and A. N. K). Articles were selected based on the inclusion and exclusion criteria detailed below. Articles were explicitly included if the 2 reviewers agreed on their suitability. However, in the case of disagreement, a third reviewer (C.W) was involved to break the tie. No date restrictions were applied. Articles with only abstracts available or those with no official English translation were excluded from the primary compilation. However, non-English articles with an official English translation from alternative sources such as Lactmed [14] were included. For each of the publications, the reviewers extracted the following information: the first author, population studied (healthy volunteers or patients), number of participants (mothers and infants), study design, dose, time postpartum, pharmacokinetic sampling information (plasma or/and breast milk and/or infant plasma, sampling times), mathematical approach for pharmacokinetic analysis, key findings, conclusions, and limitations.

### Assessment of study quality

To provide an objective measurement of quality, all selected studies were scored against a 24-item “ClinPK” checklist considered essential for reporting clinical pharmacokinetic studies [15]. This score was developed using Delphi methodology as a “best practice” scale to ensure quality of clinical pharmacokinetic studies; however, not all variables apply to the studies involving breastfeeding mother–infant pairs. Examples of criteria that did not apply to most lactation pharmacokinetic studies were the requirement for detailed reporting of approaches used for extracorporal drug removal in patients on extracorporal drug removal interventions and the criteria requiring reporting of pharmacokinetic parameters such as F (bioavailability), AUC, C_max_, and T_max_ in studies comparing drug bioavailability between 2 drugs. Additionally, the criteria that required reporting and referencing the formula used for calculating specific pharmacokinetic variables and the criteria that required reporting the specific body weight used in drug dosing were often not applicable in evaluating the lactation pharmacokinetic studies. Each article was assessed on an individual basis with the items considered not relevant excluded from the evaluation of a specific article.

### Inclusion and exclusion criteria

We included population lactation pharmacokinetic studies, i.e., studies in which the concentrations of drugs used for the treatment of malaria, tuberculosis, or NTDs were quantified in both maternal plasma and breast milk or in all 3 of maternal plasma, maternal breast milk, and infant plasma. Measuring drug concentrations in both plasma and breast milk enables the characterization of plasma-to-breast milk drug transfer, which enables linking administered drug dose to breast milk exposure. Studies that did not quantify the concentrations of these drugs in both maternal plasma and maternal breast milk or in maternal plasma, maternal breast milk, and infant plasma were excluded. Additionally, case studies were not included in our final list. The systematic review protocol is available at (https://doi.org/10.5281/zenodo.7974804).

### Results

**Fig 2:**
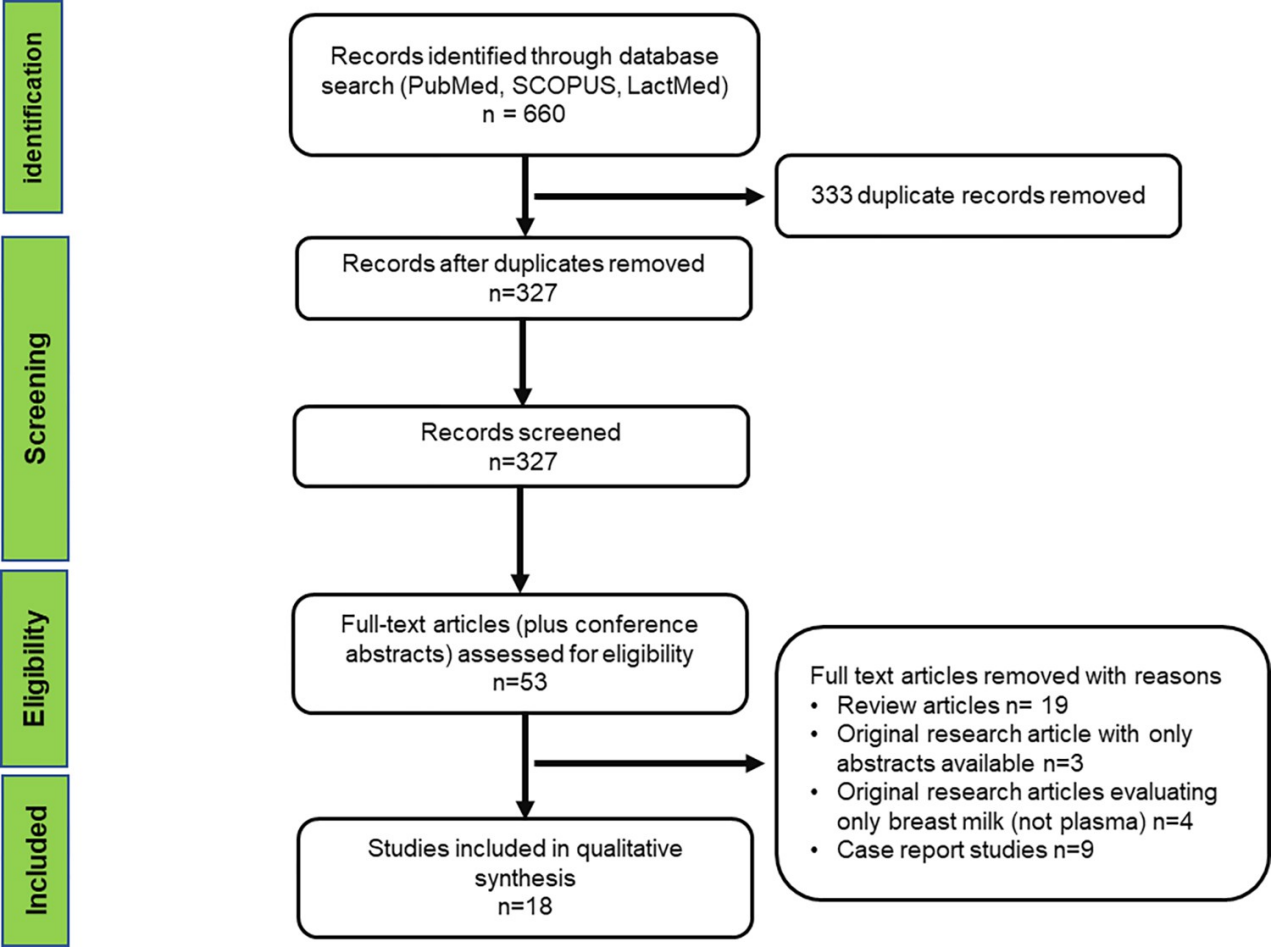
Information sources and search strategies.

**Fig 3:**
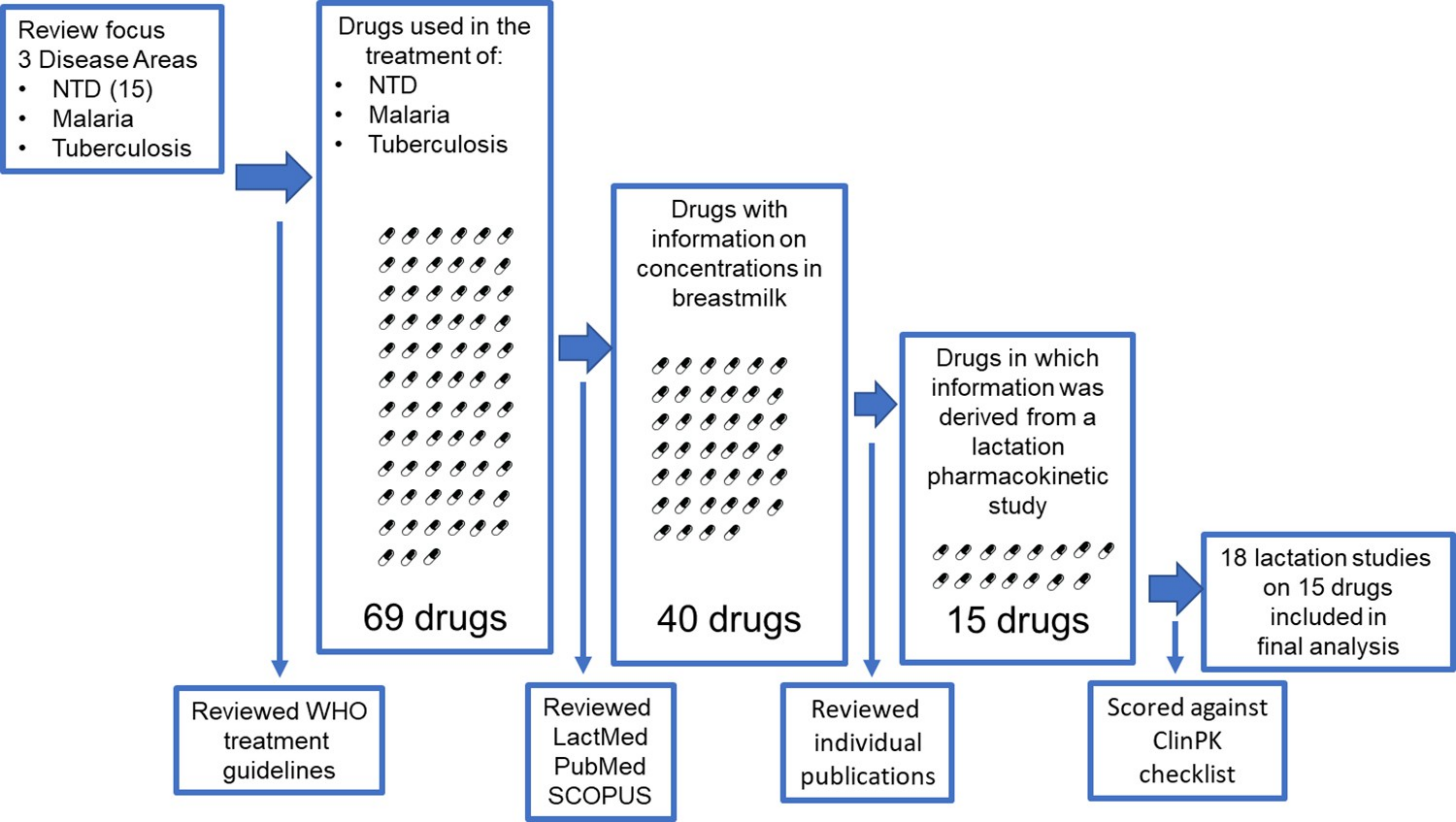
Schematic workflow undertaken for extraction of information on lactation pharmacokinetics for drugs used to treat malaria, tuberculosis, and NTDs.

A list of 69 drugs used for the treatment of malaria, tuberculosis (including RR/MDR), and NTDs was generated from the WHO treatment guidelines as summarized in **Table 1**. Of the 69 drugs listed, only 58% had any information on pharmacokinetics in breast milk available. Of those with information on pharmacokinetics in breast milk, less than half had this information derived from lactation pharmacokinetic studies, i.e., some studies only measured drug concentration in breast milk and not in plasma or took single plasma samples without time recorded relative to dose. In these, it is not possible to characterize the transfer into breast milk, and, therefore, these were not considered lactation pharmacokinetic studies. **Fig 2** shows the literature search strategy, and **Fig 3** provides a schematic of the workflow adopted in extracting the lactation pharmacokinetic data from evaluated drugs.

### Lactation pharmacokinetic studies for drugs used for the treatment of malaria, tuberculosis, and NTDs

Considering the evidence for the use of the 69 target drugs identified from WHO treatment guidelines for tuberculosis (including RR/MDR), malaria, and NTDs, only 18 clinical lactation pharmacokinetic studies, evaluating 15 different drugs, were identified. These studies reported on the following drugs: piperaquine, primaquine, chloroquine, quinine, clindamycin, mefloquine, bedaquiline, isoniazid, benznidazole, nifurtimox, albendazole, praziquantel, clofazimine, ivermectin, and azithromycin.

The number of mothers enrolled across the 18 studies was generally low, with a median of 27 mothers (range: 2 to 33). In 4 of the studies, focusing on benznidazole, nifurtimox, primaquine, and bedaquiline, breastfeeding infants were enrolled together with their mothers [27–30]. In 15 out of 18 studies (83.3%), maternal drug concentrations were measured at multiple time points after drug administration, whereas in the other 3 studies, maternal blood concentrations were measured only at a single time point after drug administration. In the 4 studies that enrolled infants, evaluation of infant blood drug concentrations was performed in 2 studies. In the study by Court and colleagues [30], limited infant plasma sampling was performed, based on 4 infants from the 13 mother–infant pairs. In the study by Gilder and colleagues [29], infant capillary blood concentrations were collected only for evaluation of the hematocrit.

Six of the 18 studies were conducted in women who were not receiving treatment for malaria, tuberculosis, or NTDs but in patients with different disease conditions or even healthy volunteers. Three of the studies that evaluated chloroquine [31,32], ivermectin [33], and praziquantel [34] were undertaken in healthy volunteers, whereas 2 studies evaluated the concentrations of azithromycin in a prophylactic setting in pregnant women about to deliver [35,36]. Eight out of the 18 studies (44.4%) reported the postpartum time points of breast milk sampling, ranging from the time of delivery to as long as 6 months across the different studies.

Across the 18 studies, 66.7% (12 out of 18) used NCA methods for pharmacokinetic evaluation, whereas 6 studies employed model-based compartmental approaches. For NCA, in studies with multiple drug concentrations across time, the most commonly estimated pharmacokinetic parameters for plasma and breast milk drug exposure were T_max_, C_max_, and AUC in plasma and breast milk [29,34,37]. Of the 12 studies employing NCA, 5 studies calculated a milk-to-plasma (M:P) ratio based on breast milk and plasma drug concentrations measured at specific time points [27,28,31,37,38]. Four studies calculated the M:P ratio as a ratio of breast milk AUC to the plasma AUC [29,32,34,39], whereas 3 studies did not report calculating a M:P ratio [34,40,41]. M:P ratios calculated from specific time points do not account for differences between plasma and breast milk concentration-time profiles, unlike those generated from AUCs, and might yield misleading findings. For studies that employed model-based compartmental analysis, the M:P ratio was calculated from model-derived breast milk and plasma AUCs [42,43] or by directly modeling the plasma-to-milk drug transfer kinetics [30,44].

### Assessment of study quality

Each of the 18 studies were individually assessed for conformity with the best practices for reporting clinical pharmacokinetic studies as recommended by the “ClinPK” guidelines. The 24 items/criteria of the “ClinPK” guidelines were not all relevant across the 18 studies. The criteria requiring that the title of the article identified the drug(s) and the patient population(s) studied was one of the 14 criteria relevant to all 18 studies, whereas the criteria requiring detailed reporting on approaches used for extracorporal drug removal in patients on extracorporal drug removal interventions was among the two not relevant for all 18 studies. The number of relevant criteria was lowest in the studies by Ogunbona and colleagues [31] and Ogbuokiri and colleagues [33], scoring 17 out of 24, and highest in the study by Sutton and colleagues [36] with 22 out of 24. The conformity to the relevant “ClinPK” criteria was highly variable across the 18 studies with the lowest scoring 8 out of 17 in the study by Ogbuokiri and colleagues [33] and the highest score of 20 out of 22 by Sutton and colleagues [36].

A summary of the various study designs, population sampling schedule, and results are reported in Table 2 below, and a further description of the findings for each of the chosen drugs is provided. Assessment of the conformity of studies with the “ClinPK” guidelines is provided in the Supporting information (S1 Table).

**Table 2:** Key findings in studies evaluating mother-to-infant transfer of antimalarials, antituberculosis, and drugs for neglected tropical diseases (NTDs).

Author	Study design, Population	Number of mothers (infants)	Dose	Time postpartum		Plasma samples (Breast milk)	PK Analysis	ClinPK Score (%)	Conclusion	Limitations
**Piperaquine** Moore et al [44].	Design: Randomized controlled trial evaluating pharmacokinetics and pharmacodynamics of dihydroartemisinin- piperaquine versus sulfadoxine pyrimethamine-piperaquine combinations for intermittent presumptive treatment Population: Pregnant and non-pregnant women (age- and community matched) with no evidence of severe malaria	27 (0)	320 mg at 0, 24, and 48 hours Standard treatment regimen	Not stated	Yes (Yes)		Compartmental	18/19 (94.7)	M:P ratio = 0.58 AID = 0.41 μg/kg/day RID = 0.004% Breast milk transfer after maternal treatment but exposure for breastfed infants appears safe.	Milk crematocrit was not estimated: Variability in breast milk concentration not evaluated. No infants enrolled
**Primaquine** Gilder et al [29].	Design: Observational study evaluation the use of standard radical primaquine regimen in preventing relapse of *P*. *vivax* malaria infection Population: Women aged ≥18 years old with a history of *P*. *vivax* infection and no prior radical cure, together with their breastfeeding healthy (no *P*. *vivax* malaria infection) infants	20 (20)	0.5 mg base/kg to nonfasted women once daily for 14 days Standard treatment	Not stated	Yes (Yes)		Non-compartmental	17/19 (89.5)	M:P ratio = 0.34 (0.12–0.64) on day 0; 0.37 (0.24–0.61) on day 13 AID: 2.98 (1.15–9.10) μg/kg on day 0; 2.58 (1.06–8.22) μg/kg RID: 0.618% (0.231–1.82) on day 0; 0.517% (0.212–1.64) on day 13 Low breast milk transfer, low chances of infant exposure. Treatment should not be withheld in breastfeeding mothers	Breast milk volume estimated rather than measured across study duration No infantpharmacokinetic data
**Chloroquine** Ogunbona et al [31].	Design: Observational study evaluating lactation pharmacokinetics and safety in breastfeeding infants when used for prophylaxis Population: Volunteer nursing mothers	11 (0)	Single oral dose of 600 mg base chloroquine Standard dose level for chemoprophylaxis	Not stated	Yes (Yes)		Non-compartmental	9/16 (56.3)	M:P ratio = 5.6 (3.8–9.0) AID = 4.4 ± 2.6 mg RID = 0.70% Breast milk concentrations were greater than plasma concentrations. The average M:P ratio at 24 hours was 6.6 ± 2.4. The peak concentration was 4.4 ± 2.6 mg/L	Limited blood/breast milk samples collected No infants enrolled
**Chloroquine** Edstein et al [32].	Design: Observational study evaluating lactation pharmacokinetics when used for prophylaxis Population: Volunteer non-breastfeeding mothers	3 (0)	Single oral dose of 600 mg base chloroquine	2–5 days postpartum	Yes (Yes)		Non-compartmental	12/18	M:P ratio = 1.96, 2.35, and 4.26 RID (%): 2.2, 2.9, 4.2 Chloroquine was excreted in breast milk at concentrations greater than that found in plasma	Limited blood/breast milk samples collected No infants enrolled
**Quinine** Phillips et al [40]	Design: Observational study evaluating the pharmacokinetics and breast milk excretion of quinine in pregnant and lactating women with falciparum malaria Population: Lactating mothers	30 (0)	10 or 20 mg/kg of quinine sulfate	Late pregnancy/Early postpartum (Exact time not stated)	Yes (Yes)		Non-compartmental	12/18	In 5 patients treated intravenously, the mean breast milk concentrations was 2.6 mg/L (range: 0.5–3.6) and mean M:P ratio was 0.21 (0.11–0.32) In 25 patients treated orally, the mean concentration was 0.5–8.0 mg/L and 0.31 (0.11–0.53) in 25 women who received oral treatment Estimated daily breast milk excretion less than 2–3 mg/day, RID (%) <1%	No infants enrolled in the study
**Mefloquine** Edstein et al [39]	Design: Observational study evaluating the breast milk excretion of mefloquine lactating women with falciparum malaria Population: Volunteer lactating mothers	2 (0)	250 mg	2–3 days postpartum	Yes (Yes)		Non-compartmental	14/18	The elimination in breast milk than in plasma The M:P ratio values of 0.16–0.13 after days and 0.27 after 56 days RID of 3.8%.	Limited number of mothers (only 2) No infants included
**Clindamycin** Steen and Rane [41]	Design: Observational study evaluating the breast milk excretion of clindamycin in women treated for puerperal anaerobic infections. Population: Lactating mother who have just given birth but not breast feeding their infants	5 (0)	150 mg	One week postpartum	Yes (Yes)		Non-compartmental	9/18	Breast milk concentrations at the end of the dosing interval ranged from <0.5 μg/mL–3.1 μg/mL. No correlation between milk and plasma concentrations at the end of the dosing interval, however a strong correlated with the area under the plasma concentration-time curve	No infant included in the study
**Bedaquiline** Court et al [30].	Design: Observational study of pharmacokinetics in pregnancy and lactation Population: Pregnant women, aged ≥18 years treated for RR-TB, together with their breastfeeding infants	13 (13)	200 mg dosed 3 times a week, after the 2-week loading dose. Standard treatment	13 women (≥28 weeks) and 6 women (6 weeks postpartum)	Yes (Yes)		Compartmental analysis	15/17 (88.2)	M:P ratio = 13.6 (% RSE = 10.1) for bedaquiline and 4.84 (RSE = 5.10) for the metabolite AID = 0.816 mg/kg/day for bedaquiline and 0.07/mg/kg/day for the metabolite RID = 66.9% The drug significantly accumulates in breast milk; breastfed infants receive mg/kg doses equivalent to maternal doses.	Unbound drug concentrations or albumin levels were not measured, possibly leading to low plasma concentrations. High rate of participant loss to follow up limiting sample size. Limited infant pharmacokinetic data, based on only 4 infants.
**Isoniazid** Singh et al [42].	Design: Observation study of lactation pharmacokinetics for mothers on standard anti tuberculosis treatment Population: Lactating women with tuberculosis, aged 18–28 years	7 (0)	300 mg single dose daily Standard treatment	Not stated	Yes (Yes)		Compartmental	15/19 (78.9)	M:P ratio = 0.89 (95% CI: 0.70–1.1) AID = 89.9 μ/kg/day (95% CI: 65.6–114) RID = 1.2% (95% CI: 0.9, 1.5) The data suggest that isoniazid therapy is safe during breastfeeding	Few mothers included in the study No infants enrolled
**Benznidazole** Garcia-Boumissen et al [27].	Design: Observational study of lactation pharmacokinetics and the risk of infant exposure Population: Lactating women with chronic Chagas disease aged 20–34 years	10 (10)	5–8 mg/kg/day twice daily for 30 days Standard treatment	Not stated	Yes (Yes)		Non-compartmental	13/17 (76.5)	M:P ratio = 0.52 (0.3–2.79) AID = 0.65 mg/kg/day RID = 12.3% (5.5–17.0) Limited drug exposure in breast milk hence may be compatible with breast feeding	No infant pharmacokinetic data Small number of infants were enrolled hence not possible to accurately evaluate adverse effects in infants
**Nifurtimox** Moroni et al [28].	Design: Observational study of lactation pharmacokinetics and safety Population: Lactating women with chronic Chagas disease age 17–36 years	10 (10)	8–12 mg/kg/day Standard treatment	Not stated	Yes (Yes)		Non-compartmental	12//18	M:P ratio = 16 (8.75–30.3) AID = 0.50 mg/kg/day (IQR: 0.20–0.69) RID = 6.70 (IQR: 2.35–7.19) Nifurtimox may be compatible with breastfeeding due to limited drug transfer into breast milk, and low overall infant exposure.	No infant pharmacokinetic data Few infants enrolled, hence not possible to accurate evaluate adverse effects
**Albendazole** Abdel-tawab et al [37].	Design: Observational study of lactation pharmacokinetics and infant exposure Population: Breastfeeding women aged 18–40 years in a lymphatic filariasis control program	33 (0)	400 mg Standard treatment	2 weeks to 6 months	Yes (Yes)		Non-compartmental	13/18 (72.2)	M:P ratio = 0.9 (0.2–6.5) for albendazole and 0.6 (0.1–1.5) for albendazole sulphoxide Albendazole (and the sulphoxide) attains low concentrations in breast milk unlikely to harm the breastfed infant	Limited (only one) maternal plasma concentration measured. No infants enrolled
**Praziquantel** Bustinduy et al [43].	Design: Randomized controlled trial for evaluating pharmacokinetics and safety of praziquantel in pregnancy Population: Lactating women infected with Schistosomiasis Japonicum	15 (0)	30 mg/kg twice over 4 hours Standard treatment with same overall dose	Not stated	Yes (Yes) 0.028		Compartmental analysis	18/20 (90)	M:P ratio = NE AID = 0.028 mg/kg/day RID = <0.05% Women in early pregnancy had increased clearance and lower exposure. Significantly lower breast milk exposure than the dose required for antischistosomal activity. Breastfeeding should not be stopped or delayed	No infants enrolled
**Praziquantel** Putter and Held [34]	Design: Randomized trial evaluating lactation pharmacokinetics of praziquantel at various dosing schedules Population: Healthy lactating women	10 (0)	50 mg/kg once (5 women); 20 mg/kg 3 times at 4-hour intervals (5 women) Standard treatment dose	Not stated	Yes (Yes)		Non-compartmental analysis (Exact method not stated)	10/16 (62.5)	The mean plasma exposure was 3.8 times the mean breast milk exposure. AID = 27.9 μg/day RID = 0.0007% Milk does not represent a deep distribution compartment but readily equilibrates with plasma. Equilibration obviously takes place by passive diffusion and not active secretion	No infants enrolled
**Clofazimine** Venkatesan et al [38].	Design: Observational study of lactation pharmacokinetics and infant exposure to clofazimine as part of multiple drug treatment Population: Female leprosy patients, aged 19–35 years	8 (0)	50 mg daily (5 women); 100 mg on alternate days (2 women); 100 mg daily (1 woman) Standard treatment	Not stated	Yes (Yes)		Non-compartmental	16/17 (94.1)	M:P ratio = 1.4 ± 0.08 at 4–6 hours after last daily dose AID = 0.199 ± 0.013 mg/kg/day RID = 22.11 ± 1.90% In spite significant breast milk exposure (infant ingestion of 0·225 mg/kg/day), discontinuation is not recommended in breastfeeding mothers till further toxicity studies	Plasma data only available at a single time point. No infants enrolled
**Ivermectin** Ogbuokiri et al [33].	Design: Controlled trial of lactation pharmacokinetics and infant exposure Population: Healthy mothers whose babies died at birth	4 (0)	150 μg/kg Standard treatment for river blindness	Not stated	Yes (Yes)		Non-compartmental	7/16 (43.8)	AID = 2.75 μg/kg RID = 2.75% Given the additional benefits of ivermectin and its tolerably, low levels in breast milk, exclusion of lactating mothers should be discontinued.	No infants included in the study. Mothers were not breastfeeding—did not sample mature milk
**Azithromycin** Sutton et al [36].	Design: randomized controlled trial evaluating perinatal pharmacokinetics of azithromycin after a single preincision administration Population: Women undergoing planned cesarean delivery at 37 weeks of gestation (or more)	30 (0)	500 mg	At delivery	Yes (Yes)		Compartmental (2-compartment model)	19/21 (90.5)	AID = 340 μg/day RID = 0.07% High and sustained breast milk concentrations obtained after a single dose. Accumulation of the drug in breast milk from simulation-based studies	No infants enrolled
**Azithromycin** Salman et al [35].	Design: randomized placebo -controlled Phase III trial of a single dose of azithromycin versus placebo for lactation pharmacokinetics and infant safety Population: Women in labor	20 (0)	2 g	Within 1 month postpartum	No (Yes)		Compartmental	16/18 (88.8)	AID = 0.7 mg/kg/day RID = 2.5% Breast milk exposure may exceed the nominal 10% safety limit in a large proportion of neonates even after a single dose	M:P ratio used was not derived from women in this study. More complex (mechanistic models) were not evaluable due to the limited data. No infants enrolled

AID, absolute daily infant dose; M:P, milk-to-plasma ratio; RID, relative infant dose.

The majority of these studies did not measure concentrations of the drugs in infant plasma limiting the characterization of the actual level of exposure in infants.

### Piperaquine

Twenty-seven women received 3 doses containing 320 mg of piperaquine (combined with dihydroartemisinin or sulfadoxine-pyrimethamine) administered at 0, 24, and 48 hours after taking a detailed medical history and physical examination of the patient. Breast milk was sampled on days 1, 2, 3 to 5, 7 to 11, and 14 to 17 after delivery. Changes in plasma and breast milk concentrations were characterized using an integrated breast milk-plasma model, with plasma concentrations described as linearly changing across time, whereas the breast milk concentrations were estimated from plasma concentrations using the M:P ratio. The estimated absolute and relative cumulative infant dose was 22 μg and 0.07%, respectively, corresponding to an absolute and RID of 0.41 μg/kg/day and 0.004%, respectively [44].

### Primaquine

The levels of primaquine in maternal blood and breast milk and blood exposure of a breastfed infant were evaluated in 20 mother–infant pairs after administering 0.5 mg base/kg of the drug daily for 14 days to non-fasted women [29]. From samples collected on days 0, 3, 7, and 13, the pharmacokinetics of the primaquine and its metabolite (carboxyprimaquine) were evaluated. A delayed distribution into breast milk was observed, with concentrations peaking 1 hour later in breast milk compared to plasma. The infant was estimated to ingest up to 0.042 mg/kg over the 14 days of treatment, corresponding to 2.98 μq/kg/day (only 0.6% of hypothetical infant daily dose of 0.5 mg/kg). The breast milk pharmacokinetics were similar on days 0 and 13, whereas those of carboxyprimaquine varied between the 2 days. The infant breast milk exposure was negligible with only a single sample, collected in one of the infants on day 7, being above the lower limit of quantification. In addition to the negligible infant exposure, no adverse events were reported in infants [29].

### Chloroquine

Two studies [31,32] evaluated the breast milk excretion of chloroquine and its major metabolite, desethylchloroquine, after a single oral dose of 600 mg. In the first study [31], 11 female lactating volunteers were evaluated. From plasma and breast milk samples collected at 0, 3, and 24 hours postdose, concentrations of chloroquine in breast milk were always greater than that in plasma, with an average M:P ratio of 6.6 ± 2.4 and 1.5 ± 0.6 for desethylchloroquine. The estimated maximum RID was 0.7%. From urine sampled in 4 of the neonates between 12 and 24 hours, chloroquine concentrations of 3.97 ± 1.6 were observed, whereas those of desethylchloroquine were 0.44 ± 0.32 [31]. In the second [32] study, 3 women were administered chloroquine 2 to 5 days postpartum, and 6 blood and breast milk samples per patient were obtained over a period of 10 days from the time of drug administration. The M:P ratio values of 1.96, 2.35, and 4.26 were calculated for chloroquine, corresponding with percentage doses excreted in breast milk of 2.2, 2.9 and 4.2. The M:P ratio values for desethylchloroquine were 0.54, 0.80, and 3.89.

### Quinine

The breast milk excretion of quinine was studied in 30 lactating mothers treated with an initial parenteral dose of 10 or 20 mg/kg of quinine sulfate [40]. The period of start of lactation spanned from within the previous 24 hours to up to 10 days ago. Five patients received between 2 and 7 doses intravenously and had mean breast milk concentrations of 2.6 mg/L (range: 0.5 to 3.6) and mean M:P ratio of 0.21 (0.11 to 0.32). Twenty-five patients who received oral treatment for 1 to 10 days had mean breast milk concentration of 0.5 to 8.0 mg/L and mean M:P ratios of 0.31 (0.11 to 0.53).

### Clindamycin

One study [41] evaluated the breast milk excretion of clindamycin. Five mothers received clindamycin 3 times a day starting immediately after childbirth. The lactation pharmacokinetics was studied within a single dosing interval after a week of treatment. The breast milk concentrations at the end of the dosing interval ranged from <0.5 μg/mL to 3.1 μg/mL. No correlation between milk and plasma concentrations was observed at the end of the dosing interval; however, the milk concentrations strongly correlated with the area under the plasma concentration-time curve. As the volumes of milk collected at the end of the doing intervals were not reported, it is not possible to compute the amounts of drug excreted and the RID.

### Mefloquine

One study evaluated the breast milk excretion of mefloquine [39]. Two women who received 250 mg of mefloquine 2 to 3 days postpartum were studied. Blood was sampled predose, at 4 hours, and 1, 2, 4, 14, 28, 42, and 56 days postdose, whereas breast milk was sampled at 0 hours, 4 hours, and 1, 2, and 4 days postdose for both women. Additionally, at 14, 28, 42, and 56 days postdose for one of the women. The breast milk elimination of mefloquine was slower than that in plasma. The M:P ratio was 0.16 and 0.13 in either of the 2 women in the first 4 days and 0.27 after 56 days. It was estimated that a 4-kg infant with a daily milk intake of 1 L would consume 0.08 mg/day, and 0.56 mg/week, corresponding to a RID of 3.8%.

### Bedaquiline

A longitudinal pharmacokinetic study of this drug was conducted in 13 pregnant women aged 30 years (interquartile range (IQR): 25 to 37) [30]. During their third trimester of pregnancy, they were dosed with 200 mg of bedaquiline 3 times a week as part of a standard regimen for treating rifampicin-resistant tuberculosis. Blood was sampled predose and at 2, 4, and 6 hours postdose in their third trimester of pregnancy (≥28 weeks) and at 6 weeks postpartum, whereas breast milk was sampled at 6 weeks postpartum. Using a population pharmacokinetic model developed to describe this data, the M:P ratio was estimated as 13.6 (% relative standard error = 10.1) for bedaquiline and 4.84 (% relative standard error = 5.10) for M2 (a metabolite of bedaquiline). The estimated infant doses were 0.816 mg/kg/day for bedaquiline and 0.07 mg/kg/day for M2. The estimated maternal daily dose of 1.22 mg/kg/day would translate into a RID of 66.9%. The bedaquiline and M2 concentrations in the breastfed infants were similar to that in maternal plasma, whereas concentrations in non-breastfed infants were detectable but lower than that in maternal plasma, i.e., less than 0.02 mg/mL for both bedaquiline and the metabolite [30]. This extensively high infant exposure (RID >10%) emphasizes the need for lactation pharmacokinetic studies.

### Isoniazid

Singh and colleagues [42] evaluated steady-state pharmacokinetic exposure in 7 women who received 300 mg of isoniazid once daily for at least 34 days as part of standard treatment for tuberculosis, also including rifampin and ethambutol. From breast milk sampled between 0 to 4 hours after a dose, the maximum concentrations were 2 to 6.7 mg/L, occurring 1 hour after the dose and the estimated exposure of a breastfed infant was 89.9 μg/kg/day corresponding to a RID of 1.2% [42]. Two earlier studies also reported the distribution of isoniazid in breast milk. In the first study, 30 lactating mothers each received a single oral dose of 200 mg of isoniazid. The average peak breast milk concentration from samples obtained between 2 and 7 hours after dose was 2.1 mg/L at 2 hours in 6 women and 0.46 mg/L at 7 hours in 3 women. In the second study, 3 mothers who received single oral doses of 300 mg and 600 mg of isoniazid reported similar time to peak drug concentrations (3 hours after dose) but higher maximum concentrations with the 600 mg compared to the 300 mg dose, i.e., a range of 9 to 10.6 mg/L versus 5.4 to 5.5 mg/L [14].

### Benznidazole

Ten lactating mothers treated with 5 to 10 mg/kg/day of benznidazole twice daily for 30 days were evaluated. From the 16 breast milk samples collected from 10 patients, the median breast milk benznidazole concentration was 3.8 mg/L. The estimated median calculated infant dose was 0.65 mg/kg daily corresponding to about 10% of the dose used to treat infants with Chagas disease and yielding a 12.3% RID [27]. Additionally, the breast milk distribution of benznidazole was also evaluated in a study developing a bioanalytical method for breast milk quantification [45]. From 8 breast milk samples from different lactating mothers treated with 5 to 10 mg/kg/day of benznidazole every 12 hours for 30 days, concentrations ranging from non-quantifiable (<0.88 mg/L) to 7.1 mg/L were observed between the fourth and 10th day. Limited clinical extrapolations can be made from this study due to the few samples collected and the lack of information on dosing times [45].

### Nifurtimox

In a single study, 10 breastfeeding mothers with chronic Chagas disease received 8 to 12 mg/kg/day of nifurtimox 3 times daily for 30 days. From 17 steady-state (days 4 to 21) breast milk concentrations obtained across all the mothers, a median concentration of 0.30 mg/L was reported. The estimated median infant daily dose was 0.5 mg/kg/day (IQR: 0.20 to 0.69) representing a median RID of 6.70%. No growth-, behavioral-, or weight-related adverse events that could be associated with nifurtimox were reported in all the infants enrolled in this study [28].

### Albendazole

The distribution of albendazole and its metabolites (sulphoxide and sulphone) in breast milk was evaluated in 20 lactating women after a single oral dose of 400 mg in a national lymphatic filariasis control program [37]. From serial breast milk samples collected from 20 patients at predose, 6, 12, 24, and 36 hours postdose, the maximum calculated concentration of albendazole sulphoxide was 352 ng/mL observed at 6.9 hours postdose. Using the average of reported breast milk concentrations, an infant milk intake of 0.15 L/kg/day would result in a dose of 0.00218 mg/kg/day and a RID of less than 0.05%. The AUC between 0 and 24 hours, and 0 and 36 hours were 3,932.2 ng•h/mL and 5,190.3 ng•h/mL, respectively [37].

### Praziquantel

In a study by Putter and Held [34], breast milk exposure of praziquantel was evaluated in 10 healthy lactating women. The amount of drug excreted in breast milk was estimated as a product of breast milk volume and associated concentrations within a specific sampling interval. In 5 women treated with a single 50 mg/kg dose, the maximum amount excreted was 8.5 μg, 2 hours postdose, and the mean of the total amount of drug excreted at 24 hours across all individuals was 27.4 μg (RID = 0.00087%). In the other 5 women treated with 3 doses, 20 mg/kg every 4 hours, maximum amounts of drug were excreted at 4 and 10 hours after the first dose, and the mean of the total amount of drug excreted at 24 hours across all individuals was 27.4 μg (RID = 0.00087%).

In another study by Bustinduy and colleagues [43], praziquantel PK was evaluated in 15 lactating women infected with *Schistosoma japonicum* after two 30 mg/kg doses of praziquantel given 3 hours apart. From serial breast milk samples obtained at 3, 6, 9, 12, 15, and 24 hours postdose, an integrated population pharmacokinetic model describing reversible transfer of the drug between plasma and breast milk was developed. The average concentration in breast milk over 24 hours was 0.185 mg/L, and the estimated daily infant intake was 0.028 mg/Kg, translating into a RID of 0.05%.

### Clofazimine

A study by Venkatesan and colleagues [38] reported the breast milk distribution of clofazimine in 8 female leprosy patients in their early lactation period (≤4 months) who were evaluated after treatment with doses between 50 mg and 100 mg daily (1 patient received 100 mg daily; 2 patients received 100 mg on alternate days; 6 patients received 50 mg daily) for periods between 1 and 18 months. From breast milk sampled between 4 and 6 hours postdose, clofazimine concentrations ranged from 0.8 to 1.7 μg/mL. The estimated infant daily dose ranged between 0.120 and 0.255 mg/kg/day representing an RID of 13.5% to 30% of the last maternal dose [38].

### Ivermectin

In the study by Ogbuokiri and colleagues [33], the distribution of ivermectin in breast milk was evaluated in healthy lactating mothers after a single dose of 150 μg/kg of ivermectin. Breast milk drug concentrations between 1 and 72 hours postdose was 9.85 ng/mL (range: 4.2 to 20.6). The maximum breast milk concentration was 14.13 ng/mL at 4 hours for 2 women, 6 and 12 hours each of the other two. The estimated daily infant breast milk consumption of ivermectin was 2.75 μg/kg, translating into a RID of 2.75%, with a limited risk of negative consequences for the infant.

### Azithromycin

Two studies [35,36] both employed model-based approaches to characterize the breast milk distribution of azithromycin administered in women just before labor. The estimated absolute and relative daily infant doses after a single maternal oral dose of 2 g were 0.7 mg/kg/day and 2.5% [35]. In women who received 500 mg within an hour before cesarean delivery, a sustained breast milk concentration was observed up to 48 hours, with a median of 1,713 ng/mL reported 30.7 hours postdose [36]. Model-based simulations further suggested that with doses (500 mg each) given once every 12 hours, one would yield steady-state concentrations after about 3 days and an exclusively breastfed infant would receive about 340 μg/day, translating into a RID of 0.07%.

### Discussion

The most remarkable finding of this review is that lactation pharmacokinetic studies have not been undertaken for the majority of drugs, even though these drugs are widely used in breastfeeding women. Of the 69 drugs identified as widely used to treat malaria, tuberculosis, and NTDs, only 15 drugs across 18 studies fit our inclusion criteria. Approximately half (58%) of the 69 drugs had some information regarding breast milk concentrations reported in literature, but in many situations, this information was not derived from lactation pharmacokinetic studies. Instead, information was drawn from other research activities including case reports or analyses done for bioanalytical assay development. This reluctance to conduct lactation pharmacokinetic studies is not new. A review by Larsen and colleagues [46] found that of 192 drugs that had some information on breast milk concentrations, only 69 drugs (from 78 studies) had sufficient data to calculate a M:P ratio. They mention that for most of the studies reporting breast milk concentrations, only a single sample had been collected, precluding the calculation of breast milk AUC (AUC_breast milk_) and the M:P ratio, which is calculated as AUC_breast milk_ / AUC_plasma_. The M:P ratio is more reliable when computed from exposure across the entire dosing interval (i.e., AUC). Considering that breast milk and plasma drug concentrations are often not parallel to each other, computing the M:P ratio based on a single time point can lead to misleading or erroneous conclusions about the distribution of the drug in breast milk [9]. In fact, a graphical comparison of plasma and breast milk data may help elucidate the dynamics of drug transfer and generate hypotheses on the underlying factors that influence maternal-to-infant transfer of drugs.

A second observation is that few mothers were enrolled in the selected studies, with a median number of 27 (range: 2 to 33). The low numbers constrain the accurate characterization of interindividual variability in the drug’s pharmacokinetics and make it difficult to identify patient factors that influence the pharmacokinetics of the drugs. Only one of the 18 studies evaluated infant plasma drug concentrations in addition to maternal blood and breast milk drug concentrations. Consequently, in 17 studies, maternal-to-infant transfer of drug (infant exposure) was estimated from the plasma and breast milk drug concentrations. While this approach offers an informed estimate, the more robust and conclusive way to ascertain infant exposure would be to evaluate concentrations in the breastfed infant. This lack of drug concentrations in infants limits the ability to directly correlate maternal plasma and breast milk concentrations with infant exposure. Caution should be made while extrapolating infant exposure from breast milk exposure. Firstly, the bioavailability from maternal breast milk can only be considered if the pharmacologically active species in breast milk is the parent compound that requires no biotransformation for activation. Secondly, the immaturity of enzymatic elimination pathways can drastically alter exposure in infants compared to adults, for example, the half-life of dolutegravir, a UGT1A1 substrate, was estimated to be approximately 4-fold longer in neonates compared to adults [47]. Thirdly, genotypic differences in drug-metabolizing enzymes between the mother and the infant may lead to differences in drug exposure between the mother and the infant. Additionally, several additional unknown factors may lead to variability in drug profiles between different infants. Nevertheless, infant exposure can still be estimated based on breast milk data alone. For example, Rodari and colleagues [48] estimated infant exposure to ivermectin based on 6 serial breast milk samples collected predose to 24 hours postdose. Quantifying maternal plasma drug concentrations provides a link between the administered dose and the ultimate process of excretion into breast milk and characterizing patient- or treatment-related factors that affect plasma concentrations will also highlight factors that affect breast milk excretion of the drug.

A third observation relates to the data analysis approaches used. Non-compartmental analysis (NCA) was used in 12 out of the 18 studies to characterize plasma and breast milk drug pharmacokinetics. Compared to compartmental analysis, NCA offers a faster means of estimating pharmacokinetic parameters such as C_max_, T_max_, elimination half-life, and the AUC [49]. However, a major drawback to its use is that it requires intensive pharmacokinetic sampling in each individual to describe the complete concentration-time profile and efficiently estimate the pharmacokinetic parameters [49]. NCA makes no assumptions about the disposition of a drug, which compartmental approaches often do. The use of NCA usually precludes the investigation of covariates (individual factors) associated with variability in the pharmacokinetics of a drug and thus its transfer from plasma to breast milk. Conversely, the compartmental analysis approach does not require as intensive sampling as NCA in each individual. However, compared to NCA, it is more computationally intensive, technically more complex, and, hence, requires a greater amount of training and depends on specific underlying assumptions on the distribution of the drug in the body to characterize the drug’s pharmacokinetics.

**Fig 4:**
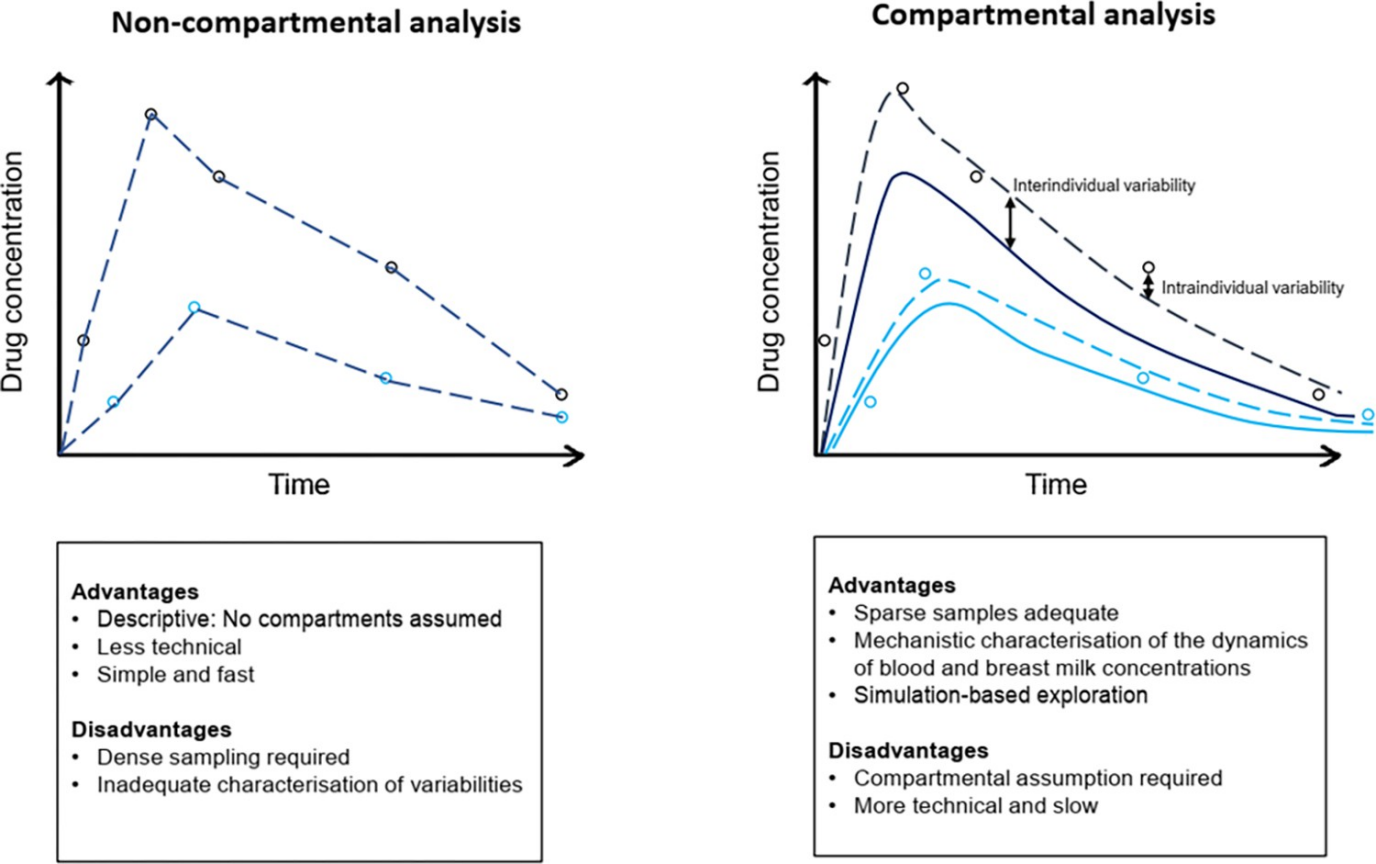
Schematic depiction of the plasma and breast milk changes in drug concentrations across time comparing non-compartmental and compartmental analysis. Left panel: Non-compartmental analysis; right panel: Compartmental analysis; open circles: Individual drug concentration data, plasma (black) and breast milk (blue); Lines: Individual drug concentration-time profiles in plasma (black) and breast milk (blue); solid lines: Population drug concentration-time profile in plasma (black) and breast milk (blue).

Nonlinear mixed-effects (NLME) modeling is the commonest compartmental analysis framework and was used to characterize the pharmacokinetics of isoniazid, azithromycin, and piperaquine [36,42,44]. NLME models simultaneously characterize typical profiles of change in drug concentrations across time within a population and the different levels of variability (within a specific patient and across different patients in a population) [50]. A key strength of NLME modeling is that it allows for robust and precise estimation of pharmacokinetic parameters even with sparse data (e.g., 3 samples per patient within a dosing interval for a study population consisting of approximately 50 patients). This reduces the burden of frequent blood draws and increases the feasibility of studies in populations where these would be difficult, for example, in pediatric or severely ill populations. Overall, compared to NCA, it reduces the logistical burden in executing the study. Additionally, these models can take various functional forms, allowing integration of knowledge on relevant physiological processes or observed trends in the data to better characterize concentration-time profiles. **Fig 4** compares non-compartmental and compartmental analyses showing the level of informativeness and the associated pros and cons.

PBPK models integrate prior diverse knowledge for a more dynamic mechanistic characterization of milk-to-plasma transfer of the drug [51,52]. To predict infant plasma drug concentrations, different infant maturation processes can be integrated in PBPK models to improve predictions.

The fourth observation was that 7 of the 18 studies were not from malaria, tuberculosis, or NTDs therapeutic areas but included patients with different disease conditions or even healthy volunteers. For example, a single 30 mg/kg (up to 2.0 g) dose of azithromycin is administered in the treatment of yaws, whereas, in this review, azithromycin was studied at 500 mg in women due for planned cesarean delivery at term [36] or at 2.0 g in women in labor [35]. Considering that drug doses and dosing schedules often differ across therapeutic areas, conclusions drawn from a pharmacokinetic study implemented in one therapeutic area may not translate to another area. Differences in doses and dosing schedules may lead to differences in pharmacokinetic exposure. This may be due to processes such as enzyme saturation, which can lead to markedly increased exposure with increasing doses or induction of enzymes leading to reduced exposure over time (single dose versus repeat dosing). Additionally, disease-related physiological changes might affect a drug’s pharmacokinetics, i.e., drug–disease interactions, which cannot be accounted for if the drugs are studied in different therapeutic areas. Furthermore, great care needs to be taken when translating results derived from studies in pregnant women to those in non-pregnant women. Pregnancy is known to cause many physiological changes that affect drug disposition including inducing drug metabolizing enzymes and drug transporters, altering amounts of proteins like albumin (which bind drugs), and increasing the volume of distribution of some drugs. As such, plasma-to-milk transfer of drugs in a pregnant or very early postpartum women might be different from that in a woman who is ≥6 weeks postpartum. On the other hand, if a drug (such as azithromycin in this case) is most likely to be used in pregnant women during or shortly before delivery, then it makes sense to determine the M:P ratio in these women to ascertain exposure in the neonate, more so if the drug has a long half-life and might be used in days/weeks leading up to delivery.

A key limitation that we observed across the studies is that many neglected to collect/report information on the postpartum times of pharmacokinetic sampling. Breast milk is a nutritionally rich matrix with several nutrients whose compositions vary with the time postpartum. It has been reported that the protein and fat concentrations in breast milk show a positive correlation with lactation from the first to the 48th month, whereas the carbohydrate concentrations show a negative correlation with lactation in the same time frame [5]. As such, fat-soluble drugs may show a higher distribution in hind milk (high fat content) compared to fore milk (low fat content) [9] since they may dissolve in the lipid droplets as they form in the alveolar epithelial cells leading to cosecretion of the drugs in breast milk.

The “ClinPK” checklist [15] was used to assess conformity of the selected studies with the minimum standards for reporting clinical pharmacology studies. Standardized guidelines facilitate transparent and complete reporting of clinical studies, thereby providing guidance to researchers conducting such studies and increasing the usability of their results by clinicians and researchers [15,53]. Clinical pharmacology trials are very diverse, sometimes not randomized, with quite different objectives, designs, and analysis methods [53]. The different guidelines for reporting clinical studies attempt to account for the diversities in study characteristics. The CONsolidated Standards Of Reporting Trials [54] is currently the most widely used criteria for reporting randomized control trials. For clinical pharmacokinetic studies that employ population-based analyses, different guidelines [55,56] have been proposed, whereas for clinical pharmacology studies, the 24-item “ClinkPK” checklist has been proposed [15]. Though there is a huge overlap in the criteria specified across the different reporting guidelines [15,54–56], divergencies result from differences in study objectives, patient inclusion, and statistical methodology. Not all the 24 items of the “ClinPK” checklist were relevant to the 18 studies. The criteria requiring detailed reporting of approaches used for extracorporal drug removal in patients on extracorporal drug removal interventions was not relevant in all 18 studies. The criteria that required a description of covariates included in a population pharmacokinetic model was only relevant in 5 out of the 18 studies. As such, each study was evaluated individually using only the relevant criteria. A lower percentage “ClinPK” score does not mean low informativeness of a study: For any 2 studies, the lack of conformity to the same number of criteria leads to a lower percentage “ClinkPK” score in the study with fewer relevant criteria.

Two recent guidelines for lactation studies have been published [57,58]. The USFDA in 2019 outlined a draft guideline for industry for conducting clinical lactation studies [57]. The guideline outlines key considerations for researchers, including ethical considerations, participant enrollment (mothers and infants), sample collection for both plasma and breast milk, data analysis, and estimation of infant exposure, and resultant adverse effects. A more recent guideline by Anderson suggested 8 major elements for reporting medication use during lactation [58]. The guideline covers pharmacokinetics and pharmacodynamics of lactation studies and seeks to identify a causal link between the pharmacokinetics of a drug and the observed effect on the infant. Pharmacodynamics elements account for 50% of the major elements in this guideline, all of which are not relevant for pharmacokinetic studies. The two guidelines can provide the initial template for further development. Criteria such as calculation of M:P ratio based on breast milk and plasma AUCs, reporting postpartum time of pharmacokinetic sampling, and infant plasma sampling would be included in a “*lactation pharmacokinetic score*.”

More clinical lactation studies have been conducted for antiretroviral drugs than any other infectious disease. In a review, Waitt and colleagues [59] reported up to 24 lactation studies covering different classes of first-line antiretroviral drugs. Of all drug classes, nucleoside reverse transcriptase inhibitors showed the highest breast milk accumulation, followed by non-nucleoside reverse transcriptase inhibitors and protease inhibitors [59]. A wide intra- and interstudy variability due to differences in pharmacokinetic sampling times, drug concentration assays, statistical methods used for data analysis, and biological differences between populations were reported, all that affect the interpretation of observed findings [59]. A more recent study characterized the breast milk transfer of the relatively newer antiretroviral drugs including dolutegravir, raltegravir, bictegravir, rilpivirine, and darunavir, showing high rilpivirine transfer, moderate to high transfer of raltegravir, and low transfer of bictegravir and dolutegravir [60]. Bictegravir and dolutegravir showed high infant concentrations despite the low breast milk concentrations observed [60], which, in the case of dolutegravir, relates to the high transplacental transfer of drug (cord: maternal blood ration of 1.2) and prolonged infant clearance due to immaturity of UGT1A1 [61].

The LactMed^®^ database provides a useful summary of clinically relevant information from lactation studies for several drugs used for treating malaria, tuberculosis, and NTDs. Lactmed summarizes data on maternal breast milk transfer and infant exposure and attempts to link this exposure with treatment outcomes on the infant, even when the data informing this overall judgment are few and sparse. This systematic review provides a broader view of different aspects of lactation studies, also including study design aspects and a summary of overall quality of the study, patient characteristics, as well as maternal plasma exposure, breast milk transfer, and infant exposure, hence providing a platform for optimizing lactation studies.

The eminent risk of unnecessary infant exposure to study drugs and possible harm from sampling blood in breastfed infants are often cited as ethical obstacles to pursuing lactation pharmacokinetic studies involving both the mother and the infants and a possible reason for not sampling from infants in most of the studies in this review. With exception of the study by Ogbuokiri and colleagues [33] that enrolled consented mothers who had lost their babies at birth, hence no risk of infant exposure, the other healthy volunteer studies [33–35, 37] enrolled breastfeeding mothers, without mention of cessation or interruption of breastfeeding during the study period. However, given that all the drugs in question are used clinically among women who live in regions of the world where breastfeeding is the only affordable, feasible, acceptable, sustainable, or safe option, a better approach is to enroll breastfeeding participants who require the medication for their own health. At the Infectious Diseases Institute (Kampala, Uganda), we have previously enrolled over 350 mother–infant pairs in lactation pharmacokinetic studies evaluating key anti-infective drugs, sampling maternal plasma, maternal breast milk, and infant plasma [61–64]. From our experience, emphasizing and clearly explaining the benefits of characterizing drug exposure of a breastfed infant to the ethics committee during study approval, and while obtaining maternal consent, has enhanced the acceptance of these studies over any presumed ethical obstacles. There is a need to share experiences and competencies between different researchers working on lactation pharmacokinetic studies to streamline the conduct of these studies to define best practices from both a bioethical and pharmacokinetic perspective.

## Conclusions

The information on plasma-to-breast milk transfer of drugs commonly used to treat malaria, tuberculosis, and NTDs, and the resultant infant exposure to these drugs through breast milk, is currently limited. The available drug-specific lactation pharmacokinetic data describe maternal blood and breast milk exposures based on data from few mothers and estimate infant drug exposure based on maternal breast milk exposure. Model-based compartmental analyses are increasingly being used to link the dynamic changes in maternal plasma concentrations with breast milk concentrations and characterize interindividual variability in drug concentration-time profiles from fewer samples, compared to non-compartmental analysis. There is an urgent need to build consensus and establish best-practice guidelines for conducting and reporting lactation pharmacokinetic studies to enhance the understanding of plasma-to-breast milk transfer of drugs, determine influential patient- and treatment-related factors, and optimize treatment in mother–infant pairs.

## Supporting information

S1 Table Assessment of clinical pharmacology lactation studies based on the ClinPK evaluation approach. (DOCX)

S2 Table PRISMA checklist. (PDF)
