# Supplementary material for: A systematic review on maternal-to-infant transfer of drugs through breast milk during the treatment of malaria, tuberculosis, and neglected tropical diseases
Source: PLoS Negl Trop Dis. 2023 Jul 13;17(7):e0011449. doi: 10.1371/journal.pntd.0011449 (PMC10343098; doi:10.1371/journal.pntd.0011449)
Supplement: S1 Table — (DOCX) [file pntd.0011449.s001.docx]

**Supplementary table 1:** Assessment of clinical pharmacology lactation studies based on the ClinPK evaluation approach

| **ClinPK check list item** | [1] | [2] | [3] | [4] | [5] | [6] | [7] | [8] | [9] | [10] | [11] | [12] | [13] | [14] | [15] | [16] | [17] | [18] |
| --- | --- | --- | --- | --- | --- | --- | --- | --- | --- | --- | --- | --- | --- | --- | --- | --- | --- | --- |
| 1 The title identifies the drug(s) and patient population(s) studied | 1 | 1 | 1 | 1 | 1 | 0 | 1 | 1 | 1 | 1 | 1 | 1 | 1 | 1 | 0 | 1 | 1 | 1 |
| 2 The abstract minimally includes the name of the drug(s) studied, the route of administration, the population in whom it was studied, and the results of the primary objective and major clinical pharmacokinetic findings | 1 | 1 | 1 | 1 | 1 | 1 | 1 | 1 | 1 | 1 | 1 | 1 | 1 | 1 | 1 | 1 | 1 | 1 |
| 3 Pharmacokinetic data (i.e., absorption, distribution, metabolism, excretion) that are known and relevant to the, drugs being studied are described | 0 | 1 | 0 | 1 | 0 | 0 | 0 | 0 | 0 | 0 | 1 | 1 | 1 | 0 | 0 | 1 | 1 | 1 |
| 4 An explanation of study rationale is provided | 1 | 1 | 0 | 1 | 0 | 1 | 1 | 1 | 1 | 1 | 0 | 0 | 1 | 1 | 1 | 1 | 0 | 0 |
| 5 Specific objectives or hypotheses are provided | 1 | 1 | 1 | 1 | 1 | 1 | 1 | 1 | 1 | 1 | 1 | 1 | 1 | 1 | 1 | 1 | 1 | 1 |
| 6 Eligibility criteria of study participants are described | 1 | 1 | 1 | 1 | 1 | 1 | 0 | 1 | 1 | 1 | 1 | 0 | 1 | 1 | 1 | 1 | 1 | 1 |
| 7 Co-administration (or lack thereof) of study drug(s) with other potentially interacting drugs or food within this study is described | 1 | 0 | 0 | 1 | 0 | 1 | 0 | NA | NA | 1 | 0 | 1 | 0 | 1 | 0 | 1 | 1 | 1 |
| 8 Drug preparation and administration characteristics including dose, route, formulation, infusion duration (if applicable) and frequency are described | 1 | 0 | 1 | 1 | 1 | 1 | 1 | NA | 1 | 1 | 1 | 1 | 1 | 1 | 1 | 1 | 1 | 1 |
| 9 Body fluid or tissue sampling (timing, frequency and storage) for quantitative drug measurement is described | 1 | 1 | 1 | 1 | 1 | 1 | 1 | 1 | 1 | 1 | 1 | 1 | 1 | 0 | 1 | 1 | 1 | 1 |
| 10 Validation of quantitative bioanalytical methods used in the study is referenced or described if applicable | 1 | 1 | 1 | 1 | 0 | 1 | 0 | 1 | 1 | 1 | 1 | 1 | 1 | 1 | 1 | 1 | 1 | 1 |
| 11 Pharmacokinetic modelling methods and software used are described, including assumptions made regarding the number of compartments and order of kinetics (zero, first or mixed order) | 0 | 0 | 1 | 1 | 0 | 0 | 0 | 0 | 1 | 1 | 1 | NA | 1 | NA | 0 | 1 | 1 | 1 |
| 12 For population pharmacokinetic studies, covariates incorporated into pharmacokinetic models are identified and described | NA | NA | NA | 1 | NA | NA | NA | NA | 1 | 1 | NA | NA | NA | NA | NA | 1 | 1 | 1 |
| 13 Formulas for calculated variable (such as creatinine clearance, body surface area, AUC [area under the curve] and adjusted body weight) are provided or referenced | NA | NA | NA | NA | NA | NA | NA | 1 | NA | 1 | NA | NA | NA | NA | NA | NA | NA | NA |
| 14 The specific body weight used in drug dosing and pharmacokinetic calculations is reported i.e. ideal body weight vs actual body weight vs adjusted body weight | 0 | NA | 0 | NA | NA | NA | 0 | NA | NA | 1 | NA | NA | NA | 0 | NA | NA | NA | NA |
| 15 Statistical methods including software used are described | 0 | 0 | 1 | 0 | 0 | 0 | 0 | 1 | 1 | 1 | 1 | 1 | 0 | 0 | 0 | 0 | 1 | 1 |
| 16 Study withdrawals or subjects lost to follow up (or lack thereof) are reported | 1 | 1 | 1 | NA | NA | NA | NA | 1 | 1 | 0 | 0 | 1 | NA | NA | NA | 0 | 1 | 0 |
| 17 Quantification of missing or excluded data is provided if applicable | 1 | 0 | 0 | NA | NA | 0 | NA | 0 | 1 | 1 | 0 | 1 | 0 | 0 | 0 | 0 | 1 | 1 |
| 18 All relevant variables that may explain inter- and intra-patient pharmacokinetic variability (including: age, sex, end-organ function, ethnicity, weight or BMI [body mass index], health status or severity of illness and pertinent co-morbidities) are provided with appropriate measures of variance | 0 | 0 | 1 | 1 | 0 | 1 | 0 | 0 | 0 | 1 | 0 | 1 | 1 | 1 | 0 | 0 | 1 | 1 |
| 19 Results of pharmacokinetic analyses are reported with appropriate measures of precision (such as range or 95% confidence intervals) | 1 | 1 | 1 | 0 | 1 | 1 | 1 | 1 | 1 | 1 | 1 | 1 | 1 | 1 | 0 | 1 | 1 | 1 |
| 20 Studies in patients receiving extracorporeal drug removal (i.e. dialysis) should report the mode of drug removal, type of filters used, duration of therapy and relevant flow rates | NA | NA | NA | NA | NA | NA | NA | NA | NA | NA | NA | NA | NA | NA | NA | NA | NA | NA |
| 21 In studies of drug bioavailability comparing two formulations of the same drug, F (bioavailability), AUC, Cmax (maximal concentration) and Tmax (time to maximal concentration) should be reported | NA | NA | NA | NA | NA | NA | NA | NA | NA | NA | NA | NA | NA | NA | NA | NA | NA | NA |
| 22 Study limitations describing potential sources of bias and imprecision where relevant should be described | 1 | 1 | 1 | 1 | 0 | 0 | 0 | 0 | 1 | 1 | 0 | 1 | 0 | 0 | 0 | 1 | 1 | 1 |
| 23 The relevance of study findings (applicability, external validity) is described | 1 | 1 | 1 | 1 | 1 | 1 | 1 | 0 | 1 | 1 | 1 | 1 | 1 | 1 | 1 | 1 | 1 | 1 |
| 24 Funding sources and conflicts of interest for the authors are also disclosed | 1 | 1 | 1 | 1 | 1 | 1 | 0 | 0 | 1 | 1 | 1 | 1 | 1 | 1 | 1 | 1 | 0 | 1 |
| **Total, ClinPK score / Score of relevant ClinPK items** | 15/19 | 13/19 | 17/20 | 16/18 | 9/17 | 12/18 | 8/17 | 11/18 | 17/19 | 20/22 | 13/19 | 16/18 | 14/18 | 12/18 | 9/18 | 16/20 | 18/20 | 18/20 |

**References**

[1] García-Bournissen F, Moroni S, Marson ME, Moscatelli G, Mastrantonio G, Bisio M, et al. Limited infant exposure to benznidazole through breast milk during maternal treatment for Chagas disease. Arch Dis Child. 2015; 100: 90–94.

[2] Moroni S, Marson ME, Moscatelli G, Mastrantonio G, Bisio M, Gonzalez N, et al. Negligible exposure to nifurtimox through breast milk during maternal treatment for Chagas Disease. PLoS Neg Trop Dis. 2019; 13(8): e0007647.

[3] Gilder ME, Hanpithakphong W, Hoglund RM, Tarning J, Win HH, Hilda N, et al. Primaquine pharmacokinetics in lactating women and breastfed infant exposures. Clinical Infectious Diseases 2018; 67(7): 1000–1007.

[4] Court R, Gausi K, Mkhize B, Wiesner L, Waitt C, Mcllleron H, et al. Bedaquiline exposure in pregnancy and breastfeeding in women with rifampicin-resistant tuberculosis. Br J Clin Pharmacol. 2022; 88: 3548-3558.

[5] Ogunbona FA, Onyyeji CO, Bolaji OO, Torimiro SEA. Excretion of chloroquine and desethylchloroquine in human milk. Br J Clin Pharmacol. 1987; 23: 473–476.

[6] Edstein MD, Veenendaal JR, Newman K, Hyslop R. Excretion of chloroquine , dapsone and pyrimethamine in human milk. Br J Clin Pharmacol. 1986; 22: 733-735

[7] Ogbuokiri JE, Ozumba BC, Okonkwo PO. Ivermectin levels in human breast milk. Eur J Clin Pharmacol. 1993; 45: 389–390.

[8] Putter J, Held F. Quantitative studies on the occurence of praziquantel in milk and plasma of lactating women. Eur J Drug Metab Pharmacokinet. 1979; 4(4):193-198.

[9] Salman S, Davis TME, Page-Sharp M, Camara B, Oluwalana C, Bojang A, et al. Pharmacokinetics of transfer of azithromycin into the breast milk of African mothers. Antimicrob Agents Chemother. 2016; 60(3): 1592–1599.

[10] Sutton AL, Acosta EP, Larson KB, Kerstner-Wood CD, Tita AT, Biggio JR. Perinatal pharmacokinetics of azithromycin for cesarean prophylaxis. Am J Obstet Gynecol. 2015; 212: 812.

[11] Abdel-Tawab AM, Bradley M, Ghazaly EA, Horton J, El-Setouhy M. Albendazole and its metabolites in the breast milk of lactating women following a single oral dose of albendazole. Br J Clin Pharmacol. 2009; 68 (5): 737–742.

[12] Venkatesan K, Mathur A, Girdhar A, Girdhar BK. Excretion of clofazimine in human milk in leprosy patients. Lep Rev. 1997; 68: 242–246.

[13] Edstein MD, Veenendaal JR, Hyslop R. Excretion of Mefloquine in Human Breast Milk. Chemotherapy 1988; 34: 165–169

[14] Phillips RE, Looareesuwan S, White NJ, Silamut K, Kietinun S, Warrell DA. Quinine pharmacokinetics and toxicity in pregnant and lactating women with falciparum malaria. Br J Clin Pharmacol. 1986; 21: 677-683.

[15] Steen B, Rane A. CLINDAMYCIN PASSAGE INTO HUMAN MILK. Br J Clin Pharmacol. 1982; 13: 661-664.

[16] Singh N, Golani A, Patel Z, Maitra A. Transfer of isoniazid from circulation to breast milk in lactating women on chronic therapy for tuberculosis. Br J Clin Pharmacol. 2008; 65 (3): 418–422.

[17] Bustinduy AL, Kolamunnage-Dona R, Mirochnick MH, Capparelli EV, Tallo V, Acosta LP. Population pharmacokinetics of praziquantel in pregnant and lactating Filipino women infected with Schistosoma japonicum. Antimicrob Agents Chemother. 2020; 64(9): e00566-20; https://doi.org/10.1128/AAC.00566-20.

[18] Moore BR, Salman S, Benjamin J, Page-Sharp M, Yadi G, Batty KT, Siba PM, Mueller I. Pharmacokinetics of piperaquine transfer into the breast milk of Melanesian mothers. Antimicrob Agents Chemother. 2015; 59 (7): 4272-4278
